# Convergent and divergent selection drive plumage evolution in woodpeckers

**DOI:** 10.1038/s41467-019-14006-3

**Published:** 2020-01-09

**Authors:** Gregory F. Grether

**Affiliations:** Department of Ecology & Evolutionary Biology, University of California, Los Angeles, Los Angeles, CA 90095-1606 USA

**Keywords:** Coevolution, Mimicry, Phylogenetics

**Arising from** E. Miller, et al. *Nature Communications* 10.1038/s41467-019-09721-w

Large phylogenetic comparative studies are becoming increasingly common because of advances in computational resources and the availability of massive, online databases. Fully interpreting the results of such studies can be challenging, however. Here I draw attention to an oversight in Miller et al.’s^1^ otherwise impressive paper on plumage evolution in woodpeckers. Their results are even more consistent with evolutionary theory than conveyed in the paper and provide striking evidence that woodpecker plumage has been shaped in multiple directions by natural selection.

Divergent character displacement has been one of the cornerstone ideas in evolutionary biology since *The Origin of Species*^2^. Closely related species are generally expected to diverge from each other through natural selection in ways that reduce resource competition and costly interactions such as reproductive interference and aggression^3–5^. But under special circumstances, resource competition and reproductive interference might instead cause species to converge phenotypically^5–7^. There are several known ways this could occur^6–9^, all of which can be classified as a type of competitive mimicry^10^.

Some species of woodpeckers are remarkably similar in plumage, and it has long been suspected that at least some such cases are examples of competitive mimicry^7,8,10^. Miller et al.^1^ assembled a dataset that includes all 230 species of woodpeckers in the world and found, after controlling for a multitude of other factors, that plumage convergence in this clade is strongly associated with geographic range overlap. This is an exciting finding because it substantiates the competitive mimicry hypothesis, but a key figure in their paper reveals an even stronger association between range overlap and plumage divergence.

Specifically, the negative correlation just above 0.25 in Fig. 4 tells us that species pairs with a plumage dissimilarity of 0.2–0.3 have greater range dissimilarity (less geographic overlap) than species pairs in the other plumage dissimilarity bins (0–0.1, 0.1–0.2, 0.3–0.4, etc.). In other words, woodpecker species that are similar—but not highly similar—in coloration are rarely found together in sympatry. This is exactly what we would expect to see if some species have converged in plumage to resemble each other closely while other sympatric species pairs that once were more similar in plumage have undergone divergent character displacement. Species pairs in the other bins (0.3–0.4, 0.4–0.5, etc.), which are moderately to very dissimilar in plumage, would not be expected to show either character displacement pattern because they evidently have not converged in plumage and are already dissimilar enough that there would be no benefit to diverging any further. Species sorting due to competitive or sexual exclusion^4,11,12^ might also have contributed to this pattern.

Referring to the downward spike in Fig. 4, Miller et al.^1^ consider that it “could be interpreted as evidence that allopatry in and of itself drives plumage divergence between somewhat similar looking species pairs” but conclude that “this seems biologically implausible.” By contrast, my interpretation is they found clear evidence for accelerated divergence in sympatry exactly where we would expect to find it if divergent character displacement has occurred in woodpeckers. The possible mechanisms driving plumage divergence in woodpeckers include selection against interspecific mating (i.e., reproductive character displacement)^3,4^ and selection against interspecific aggression (i.e., divergent agonistic character displacement)^5^.

In this light, Miller et al.’s^1^ study is truly a landmark paper because it shows that it is possible to partition a large dataset in such a way as to detect patterns caused by opposing evolutionary processes in different subgroups of species within a larger clade. In most comparative studies, only one modal trend is discernible and taxa that deviate from that trend are viewed as outliers. By partitioning their data into bins of phenotypic dissimilarity, Miller et al.^1^ have provided the first concrete evidence that convergent and divergent selection have both shaped trait evolution in a single avian clade. Determining why some sympatric woodpecker species have converged in plumage while others have diverged will require intensive ecological and behavioral research. Miller et al.’s^1^ contribution will likely inspire many future studies.
